# Serosurveillance and participatory disease surveillance of Peste des Petits Ruminants (PPR) in Georgia and Armenia in 2019–2020

**DOI:** 10.1186/s12917-026-05781-5

**Published:** 2026-09-04

**Authors:** Jerome N. Baron, Nino G. Vepkhvadze, Tigran Markosyan, Ana Gulbani, Tea Enukidze, Vasili Basiladze, Tengiz Chaligava, Demna Khelaia, Natia Kartskhia, Tengiz Kurashvili, Satenik Kharatyan, Khachik Sargsyan, Ketevan Goginashvili, Maka Kokhreidze, Tamar Tigilauri, Natia Toklikishvili, Marina Turmanidze, Eliso Mamisashvili, Karl Ståhl, Guillermo Risatti, Erika Chenais

**Affiliations:** 1 Heidelberg Planetary Health Hub, Heidelberg Institute of Global Health (HIGH), Heidelberg University Hospital (UKHD), Heidelberg, Germany; 2 LEPL State Laboratory of Agriculture (SLA), Tbilisi, Georgia; 3 Scientific Centre for Risk Assessment and Analysis in Food Safety Area CJSC, Yerevan, Armenia; 4 LEPL National Food Agency (NFA), Tbilisi, Georgia; 5 Department of Epidemiology, Swedish Veterinary Agency (SVA), Uppsala, Sweden; 6 Connecticut Veterinary Diagnostic Medical Laboratory, Department of Pathobiology and Veterinary Science, College of Agriculture, Health and Natural Resources, University of Connecticut, Storrs, CT USA; 7 Swedish University of Agricultural Sciences (SLU), Uppsala, Sweden

**Keywords:** Disease freedom, Small ruminants, Caucasus, Epidemiology, Morbillivirus

## Abstract

Peste des petits ruminants (PPR) is a severe viral disease of small ruminants, endemic in Asia, the Middle East, and Africa, and a WOAH and FAO target for eradication. In the last decade, PPR emerged in countries in the Caucasus and Europe. Georgia’s first outbreak in 2016 led to multi-year vaccination campaigns in Georgia and neighbouring, PPR-free, Armenia. This was followed by serological surveillance campaigns in 2019–2020 in both countries. In addition, in 2019, participatory diseases surveillance including interviews and focus-group discussions were conducted in four provinces of Georgia. Nearly 6,600 animals were sampled across seven provinces in Georgia and three provinces in Armenia in 2019 and 2020. For each animal, serum and blood samples were collected to test for antibodies and viral RNA, respectively. The participatory disease surveillance identified symptoms compatible with a PPR outbreak in January 2016 near Tbilisi, but not in other locations or time periods. All blood samples were negative for viral RNA, and 23 serum samples were sero-positive but non-clustered in space and time. In the absence of evidence of PPR virus circulation or disease occurrence, we calculated the probability of disease freedom at the time of sampling in both countries, as well as projections for disease freedom under multiple hypothetical sampling scenarios. With a design prevalence and probability of introduction of 5%, we calculated a probability of freedom of 70.8% in Armenia and 73.8% in Georgia for 2021. In the absence of further sampling, these probabilities dropped in subsequent years due to a high risk of re-introduction from PPR-endemic neighbouring countries, exemplified by a novel introduction of PPR in Georgia in 2024. This showcases the need for continued investment in surveillance as well as transboundary collaboration between countries with different disease statuses.

**Supplementary Information:**

The online version contains supplementary material available at https://doi.org/10.1186/s12917-026-05781-5.

## Introduction

Peste des petits ruminants (PPR) is a severe and highly contagious viral disease affecting both wild and domestic small ruminants, causing significant animal suffering and economic losses, while also threatening food security and the livelihoods of farmers in regions heavily dependent on sheep and goat farming. The disease is caused by PPR virus (PPRV), also known as small ruminant morbillivirus (SRM), from the family *Paramyxoviridae*, genus *Morbillivirus*. PPR is often manifested as an acute disease with very high morbidity and mortality [1, 2]. It is endemic across Africa, the Middle East, and Asia. PPR is a WOAH (World Organisation for Animal Health) reportable disease [2]. Together with FAO (Food and Agriculture Organization of the United Nations), WOAH has set a target for eradication of PPR by 2030 and published a four-stage strategy for surveillance, control, and eradication which allows participating countries to eventually attain disease-free status [3].

More recently, PPR has been assessed as an emerging threat in Europe [4]. Since 2016, PPR has been introduced into Georgia in 2016, Bulgaria in 2018, Greece and Romania in 2024, and Hungary and Albania in 2025 [5–15]. Armenia and Georgia are located in the Caucasus region. In and around the Caucasus, Russia and Azerbaijan have PPR WOAH disease free status and Armenia is considered “historically free” (WOAH stage 4). However, Iran and Turkey are considered endemic with PPR [6, 16]. Iran reported a first outbreak in 1995 which was followed by numerous outbreaks in domestic and wild small ruminants [6, 17–22]. Similarly, Turkey first reported PPR in 1996, with additional outbreaks being reported in 2000 [6, 23–31]. In proximity to the Caucasus region, Iraq has also suffered outbreaks, notably in the Kurdistan region in the North, with both domestic and wild ruminants infected [32–35]. In Northern Iraq and Iran, in close proximity to the borders with the Caucasus countries, two wild ruminant species susceptible to PPR are present: wild goats (*Capra aegagrus*) and wild sheep (*Ovis orientalis*) [19, 25, 32, 36]. The habitat ranges of both species include Armenia and Georgia [37].

**Fig. 1:**
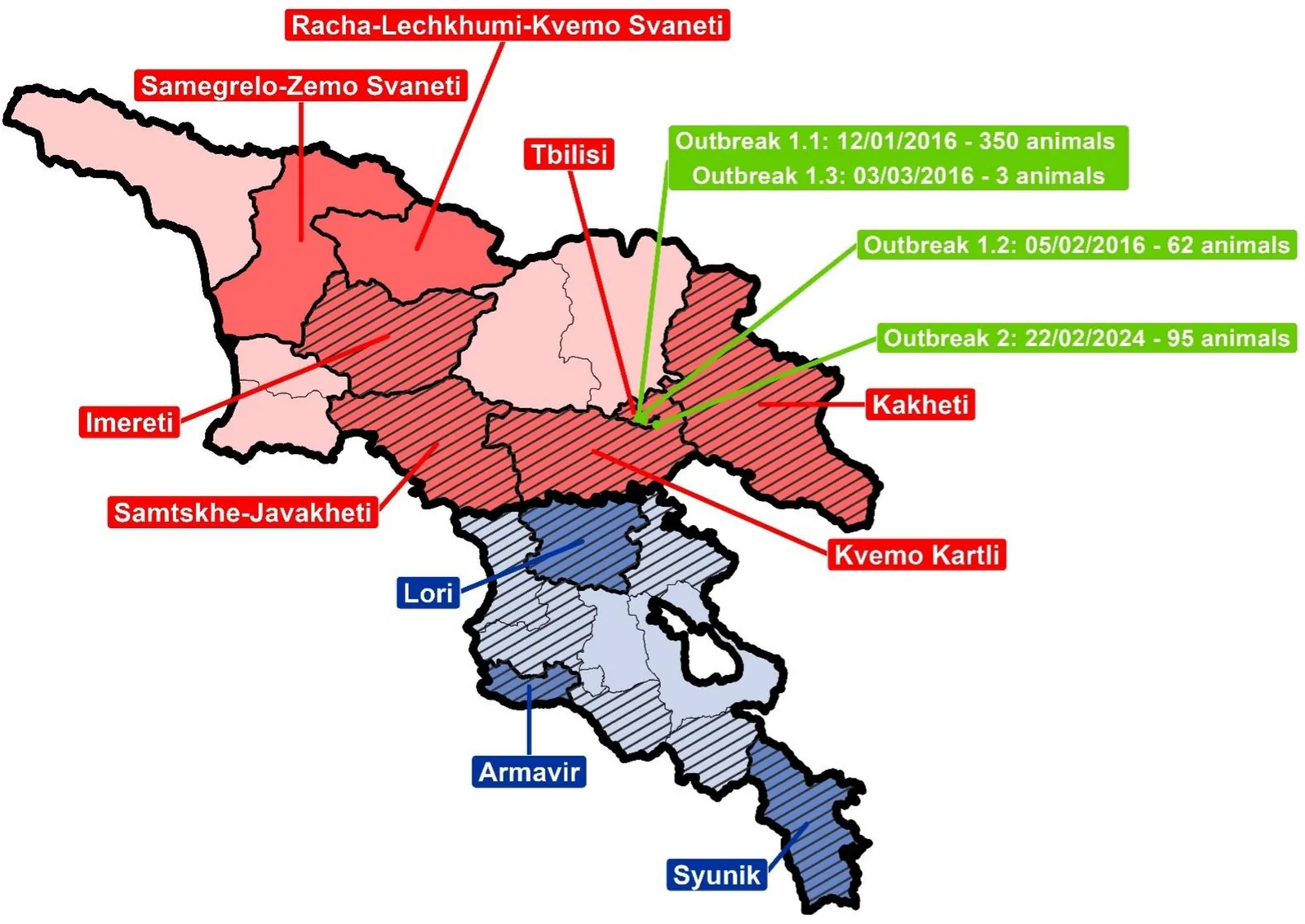
Regions in Georgia (red) and provinces in Armenia (blue) in which sero-surveillance was conducted in 2019–2020. Darker colours represent selected regions/provinces in which sampling was conducted. Regions with hashed lines represent areas where vaccination was conducted

In January 2016, PPR was detected in Georgia for the first time, affecting sheep in the region of Tbilisi (Fig. 1) [5, 38, 39]. A vaccination campaign was initiated using a modified live PPRV vaccine produced by the Federal Centre for Animal Health (ARRIAH) in the Russian Federation. Sheep and goat herds were vaccinated in the regions of Kakheti, Samtskhe-Javakheti, Kvemo Kartli, Imereti and Tbilisi which were areas affected by or in close proximity to the outbreak. A number of other control measures were applied: quarantine of affected farms, movement restrictions, culling and disposal of infected animals, and disinfection of affected farms. The outbreak was resolved by February 2016, with a total of 415 animals affected and a total of 1.65 million animals being vaccinated/revaccinated in 2016, another 1.91 million vaccinations/revaccinations up to 2021, and with the goal to stop vaccination in 2022 [6]. The re-introduction of PPR in 2024, and the subsequent control measures, put Georgia in WOAH’s stage 3 (eradication stage) [16]. Following the outbreak in Georgia in 2016, Armenia vaccinated about 407,000 animals, using the vaccine mentioned above, in provinces bordering Georgia, Turkey, and Iran considered at high risk of introduction during the period 2016–2017 [6]. In the aftermath of the outbreak in Georgia in 2016, passive and active surveillance activities were undertaken in both Georgia and Armenia. A key component of this effort included serological sampling undertaken in parallel in both countries in 2019–2020 to determine seroprevalence, or in the absence of evidence of PPRV circulation, to demonstrate possible freedom of PPR. In Georgia this was accompanied by an interview study using participatory disease surveillance techniques involving small ruminant holders in focus group discussions (FGD) concerning small ruminant management, health priorities, and problems [40].

In February 2024 a second outbreak of PPR was suspected in Georgia. This occurred in a herd of 1700 sheep in the Gardabani municipality of Kvemo Kartli region, close to the location of the 2016 outbreak in Tbilisi. Ninety-five animals were affected, of which 77 died and 18 were euthanized [41]. PPRV presence in spleen, liver, kidney, and blood samples was confirmed by the Legal Entity of Public Law (LEPL) State Laboratory of Agriculture (SLA) through RT-PCR in March. The outbreak was considered resolved in May 2024. The viral RNA was sequenced and identified this outbreak as being caused by a virus from the same lineage IV as the 2016 outbreak. However, analysis of the sequences showed higher similarity to sequences from Chinese and Mongolian strains than to the strain observed in Georgia in 2016. This suggests a novel introduction rather than a resurgence of the 2016 outbreak. In response to the outbreak, both vaccination and large-scale testing were resumed.

In this study we aim to estimate the probability of freedom from PPR in Armenia and Georgia prior to the outbreak in 2024, based on serological sampling, disease freedom calculation and participatory disease surveillance. Despite the new outbreak, disease freedom calculations help to retrospectively evaluate the power of the prior surveillance campaign and guide the requirements for future surveillance programs in the region.

## Materials and methods

### Sero-surveillance and freedom of disease calculations

#### Study area and sampling strategy

The sampling was designed as a two-stage clustered risk-based surveillance approach to quantify sero-prevalence and virus circulation in high-risk areas of both Armenia and Georgia. Disease freedom calculations were not considered when designing the sampling strategy. Thus, the sampling targeted specific higher-risk areas as opposed to a national coverage. The sampling frame was designed around the first three levels of administrative divisions which are the province, municipality, and community in Armenia, and the region, municipality, and village in Georgia. For simplicity we will use the Armenian terminology from this point on in the text. The three provinces of Lori, Armavir, and Syunik were selected for sampling in Armenia, and the seven provinces of Samegrelo Zemo Svaneti, Imereti, Racha Lechkhum, Kvemo Svaneti, Samtskhe-Javakheti, Kvemo Kartli, Kakheti, and Tbilisi were selected in Georgia (Fig. 1). In Georgia the four provinces of Kakheti, Kvemo Kartli, Samtskhe-Javakheti, and Tbilisi were selected based on their high density in small ruminants and level of risk concerning PPR which was assessed as higher than other parts of the country [5, 38]. The study area selection is described in detail in a prior interview study focusing on attitudes and management of small ruminants in relation to infectious diseases such as PPR [40]. In this study, PPRV risk was defined based on expert opinion, taking into consideration the location of the 2016 PPR outbreak in the Tbilisi area, migration routes, and border provinces with Turkey where PPR is endemic in both domestic and wild ruminants [4, 18, 19, 21, 29, 30]. These provinces were the focus of control measures, including vaccination, in the aftermath of the 2016 outbreak. The provinces of Samegrelo Zemo Svaneti, Imereti, and Racha Lechkhum Kvemo Svaneti in western Georgia where vaccination did not occur were also sampled (in smaller numbers), to evaluate antibody circulation in the absence of vaccination. In Armenia, the selection of provinces was guided by proximity to areas where PPR had been detected in the neighbouring countries. Lori borders Georgia, near the outbreak location in Tbilisi; Armavir borders endemic areas in Turkey; and Syunik borders endemic areas in Iran. The smallest administrative unit used for sampling was the community. For both countries, the sample size was calculated using Epitools with the following parameters: a fixed sample size of 20 animals per cluster, a conservative design prevalence of 50%, a number of clusters of 800, a cluster size of 150, and intra-cluster variation of 0.2, and a between cluster variation of 0.05. Intra-cluster variation can have a wide range of values in veterinary infectious diseases depending on the disease and the context, ranging from 0.06 to 0.42 [42]. A median of 0.029 for PPRV has been identified in a study in Ethiopia, but with values as high as 0.5 or more, leading us to choose a more conservative value of 0.2 [43]. This yielded a sample size of 77 clusters and 1540 animals per year, which was rounded to 80 clusters and 1600 in Georgia and 82 clusters and 1640 animals in Armenia based on the distribution of clusters within municipalities and communities proportional to population to avoid cluster fractions.

In Armenia, the sampling cluster was defined as the community. Communities were selected for sampling in all municipalities of the three selected provinces (12 in total). Out of 319 communities in the selected provinces, 82 were selected using a random number generator (supplementary table SP1). A community could only be selected for sampling if it had at least 50 animals under six months of age and had at least three herds to sample from. Thus 17 communities sampled in 2019 were not sampled again in 2020 and were replaced by 17 others as they no longer met the sampling criteria in 2020 with herd sizes and composition changing from year to year. Sampling clusters were distributed between regions proportionally to the population, giving 36 clusters in both Armavir and Syunik, and 10 communities in Lori. The 20 animals were then randomly sampled in each selected community, for a total of 1640 animals per year. No more than five animals were selected within any single herd of a given community. The total amount of communities sampled was 99 over two years, for a total of 3280 animals.

In Georgia, the sampling cluster was defined as the herd, so a given community could contain multiple clusters. 80 clusters were selected for sampling. However, contrary to Armenia, not all municipalities in the sampled provinces were covered by sampling. Twelve out of 47 municipalities and 36 out of 556 communities were selected in a first step following two criteria: seasonal migration and population of small ruminants with the help of WinEpi in two stages [44] (supplementary table SP2). The 12 selected municipalities contained 251 communities which were listed and used as a sampling frame for random selection using the random number generator from Epitools [45]. Communities with a population of less than 200 sheep and goats were excluded. The 80 clusters were then selected within the 36 communities, proportionally to the community population size, with 20 animals to be sampled per cluster and year, for a total of 1600 animals per year. The three non-vaccinated Western provinces, as well as urban Tbilisi were only allocated two sampling clusters each as they had smaller small ruminant populations. However, as with Armenia, eligibility changes lead to differences between years: in practice, samples were collected from 13 municipalities in 2019 and 15 in 2020, for a total of 22 municipalities. All animals were obtained from private farmers.

#### Sampling procedure

Blood and serum were collected from sheep and goats from the anterior *vena cava* or from the *vena jugularis* using the Vacutainer^®^ system (BD, Franklin Lakes, NJ, USA) tubes, needles, and holders. Complete blood (4 ml), used for detecting PPRV, was collected in plastic 13 × 75 mm tubes containing EDTA. Serum samples (4 ml), collected for detecting antibodies against PPRV, were collected in plastic 13 × 75 mm tubes. Blood was drawn using 21 g x 1.5-inch needles. Collection tubes were labelled and kept refrigerated during transport to the Reference Laboratory for Especially Dangerous Pathogens of the Republican Veterinary and Phytosanitary Laboratory Services Centre of the Food Safety Inspection Body of Armenia and the State Laboratory of Agriculture of Georgia for testing. At the laboratories, sera were separated. Sera and blood samples were kept refrigerated until testing. All animals were tested for both antibody detection and viral RNA detection.

The study areas included areas where herds had received vaccination. To avoid vaccinated animals or animals with maternal antibodies, the inclusion criteria was to sample unvaccinated animals of three to six months of age [46, 47].

#### Antibody detection (ELISA)

Sera were separated by centrifugation. Three 1.2 ml aliquots for each collected serum were stored at -20 °C until processing. A competitive ELISA test ID Screen PPR competition (ID vet, France) was used for detecting antibodies against the nucleoprotein (N) of PPRV. The test and interpretation of the result was performed according to the manufacturer’s instructions. The test has a sensitivity of 94.5% and a specificity of 99.4% [48, 49].

#### RNA detection (RT-qPCR)

Total RNA was extracted from 140 µl of transport media containing whole blood samples using the RNeasy kit (Qiagen, Stanford, CA, USA) and eluted in 80 µl of Tris-EDTA buffer according to the manufacturer’s instructions. RNA was stored at -80 °C for further analysis. PPRV RNA extraction was performed according to the manufacturer’s instruction [50]. We used a rapid detection assay based on Peste des Petit Ruminants Virus Real-Time qPCR Assay - The Tetracore kit cat # TC-9032-064. The RT-PCR cycle conditions program was performed according to the manufacturer’s instructions. The assays target the PPRV N gene using forward primer AGAGTTCAATATGTTRTTAGCCTCCAT, reverse primer TTCCCCARTCACTCTYCTTTGT and FAM-5-CACCGGAYACKGCAGCTGACTCAGAA-TAMRA probe used in combination with Quantabio qScript™ XLT One-Step RT-qPCR Tough Mix highly sensitive master mix [51]. This assay has a sensitivity of 90.5% and a specificity of 100% [52].

#### Disease freedom calculations

To assess the confidence in the sustained absence of PPR in Georgia and Armenia following eradication (Georgia) and testing (Georgia and Armenia), disease freedom calculations were conducted. Disease freedom calculations are an application of Bayes theorem of conditional probabilities that aim to estimate the probability of a disease being absent given a number of known priors relating to sampling outcomes and testing parameters [53]. This method can be applied to multiple stages of sampling as is our case here [54]. Calculations were conducted using the R package “freedom” for the years 2019 and 2020 [55, 56]. As not all municipalities and provinces were sampled, non-sampled areas were excluded from calculations, and the results are only valid for the areas sampled. Sampling data was recorded and aggregated at the community level in Armenia and municipality level in Georgia, which are also the respective levels at which small ruminant census data was provided for both countries. These calculations were done in four overall steps described below: (1) calculation of the surveillance sensitivity of the smallest epidemiological unit, (2) calculations of prior and posterior probabilities of disease freedom at the unit level, (3) extrapolation of calculations at the national level, (4) evaluation of the long-term impact of a potential sustained surveillance system. Parameters are summarized in Table 1. We assumed that all sampled animals were disease negative for the disease freedom calculations, despite some being seropositive. This is because seropositive animals were negative for viral RNA and showed no clinical signs in follow-up investigations.

**Table 1:** Summary of parameters used in disease freedom calculations

Parameter	Abbreviations	Value or equation
Step 1		
Test sensitivity	Sn	90%
Design prevalence	DP	1% and 5%
Sample size in unit z	n_z_	Variable
Population in unit z	M_z_	Variable
Surveillance sensitivity in unit z	SN_z_	$\:1-{(1-\frac{{n}_{z}\times\:Sn}{{M}_{z}})}^{DP\times\:{M}_{z}}$
Step 2		
Probability of introduction (national)	P_intro_	1%, 5%, and 10%
Number of units	Z	99 in Armenia, 22 in Georgia
Prior probability of disease freedom in year y in unit z	PrDF_zy_	$\:{PoDF}_{zy-1}\times\:(1-\frac{{P}_{intro}}{Z})$
Posterior probability of disease freedom in year y in unit z	PoDF_zy_	$\:\frac{{PrDF}_{zy}}{1-(\left(1-{PrDF}_{zy}\right)\times\:{SN}_{z}}$
Step 3		
Between unit design prevalence	BP	$\:\frac{1}{Z}$
System wide sensitivity	SySn	$\:1-\prod\:_{z=1}^{Z}{(1-\frac{{Sn}_{z}}{Z})}^{BP\times\:Z}$
System prior probability of disease freedom in year y	PrDF_y_	$\:{PoDF}_{y-1}\times\:(1-{P}_{intro})$
System posterior probability of disease freedom in year y	PoDF_y_	$\:\frac{{PrDF}_{y}}{1-(\left(1-{PrDF}_{y}\right)\times\:{SySn}_{y}}$

The first step was to calculate the yearly surveillance sensitivity in the smallest epidemiological unit based on the test sensitivity and the number of animals sampled in this unit. Usually, this calculation is done at the herd level, however since the smallest group resolution recorded in the sampling data was the community in Armenia and municipality in Georgia, these were the units at which the calculations were conducted. These will be referred to as units from this point on. The assumption of finite population was applied to both Armenia and Georgia, using provided population data for each unit. Typically, this method leverages the differences in expected prevalence in different animal subgroups with different risk profiles to adjust the herd level sensitivity using a decision tree approach which highlights the improved sensitivity obtained from risk-based surveillance [56]. However, in this case, we had no information on risk groups, so a simplified calculation with a single risk group was applied. Parameters for this step included the number of samples per unit and year, the estimated test sensitivity, and the design prevalence. The value used for test sensitivity was 90% [48, 49]. Two values were tested for design prevalence: a value of 5%, as well as a more conservative value of 1%. The latter was chosen as seroprevalence results around this value has been observed in other non-endemic areas in the region with re-occurring spontaneous outbreaks [20].

Next, prior and posterior probabilities of disease introduction at the unit level were calculated for 2019 and 2020, using the unit level sensitivity (calculated in step 1), the estimated probability of introduction, and temporal discounting. Prior and posterior probabilities of freedom refer to the values before and after sampling is conducted for a given year, with the prior probability of one year being obtained by discounting the previous year’s posterior probability by the yearly probability of introduction [56, 57]. For 2019, with no prior sampling, the conservative value of 50% was used for the prior probability. For subsequent year, the prior probability is obtained using the posterior probability of the previous year and the probability of introduction. As described in the introduction, PPR is endemic in the neighbouring countries Turkey and Iran, and the region is considered highly suitable for PPR based on modelling [58]. We thus made the assumption that there is a high risk of cross-border introduction of PPR to both Armenia and Georgia. In the absence of a full quantitative risk assessment on the introduction of PPR into Georgia or Armenia, two values were initially selected for probability of introduction. A first moderate value of 1% (one introduction every 100 years), as well as high value of 5% (one introduction every 20 years) was used. Finally, as PPR was identified again in Georgia in March of 2024, eight years after the previous outbreak, a value of 10% (one introduction in 10 years) was added to the calculations [41]. The values used for introduction risk were assumed at the national level, and for both countries, as we had no data to provide more geographically precise distribution of the risk of introduction, and both countries share similarly endemic neighbours. To use the national probability of introduction in the unit level calculations it was divided by the number of units sampled (99 in Armenia and 22 in Georgia) [59, 60].

Nation-wide extrapolations were then conducted. National level sensitivity requires consideration of the sensitivity values for the units within the country, the number of units sampled within the country, and the between-unit prevalence. This later parameter was selected based on the ability of the sampling frame to detect disease presence in at least one unit at the given design prevalence to confirm absence at the country level. At the national level, this was a value of one in 22 units (4.55%) in Georgia and one in 99 (1.01%) in Armenia. Disease freedom probabilities were calculated in the same fashion as they were at the unit level, but with the national sensitivities replacing the unit ones, and the probability of introduction being the national one.

To evaluate the long-term impact of a potential sustained surveillance system, the equilibrium posterior probability of disease freedom and the time to reach 90%, 95%, and 99% freedom, was calculated for each combination of the different values of design prevalence and probability of introduction presented in Table 1. The equilibrium probability of disease freedom represents the maximum value that a given surveillance system would achieve if sampling continued as defined infinitely. For this calculation, we evaluated the impact of the current situation (no more sampling after 2020) and various sampling scenarios (25, 50, and 100 animals per year in each unit). This was done using the R package ‘epiR’ [61]. All analysis, graphs, and maps were conducted using R version 4.2.2 [62].

### Participatory disease surveillance

To further validate the results while using the power of local knowledge to strengthen the evidence, an interview study using participatory disease surveillance techniques was conducted. Due to time and budget constraints this was only done in Georgia. The data was collected in June 2019 as part of a study that has been previously published [40]. The study spanned broader subjects of small ruminant management and health, only the questions concerning PPR are included here. Participant selection and data collection are described in the previous publication and summarized here in brief. Using the same risk-based criteria as described in Study area and sampling strategy section, four provinces were selected for inclusion: Kakheti, Kvemo Kartli, Samtskhe-Javakheti and Tbilisi. In each study province one, two or three municipalities, and in each municipality one or two communities were purposively selected to include study locations with a high density of small ruminants, as well as differences in geography and small ruminant husbandry traditions. In each selected community, two FGDs were held. Participants were selected on the basis of purposive sampling strategies [63]. Additionally, individual interviews were performed to reach a broader inclusion from groups of people that were underrepresented in the FGDs. These categories included for example people having fewer animals, women, shepherds and traders.

Interviews were guided by a pre-defined topic guide (supplementary document 1) outlining broad topic areas while remaining open to inclusion of additional topics by participants. The topic guide included several topics around small ruminant management, health, priorities and disease experiences. This study reports the results concerning PPR, stemming from the topic “Local disease panorama and disease priorities”, other results have been previously reported [40].

In the beginning of each interview the research team informed about the study and its objectives, and all respondents were asked for consent (including for audio recordings and photos) and informed that they could refuse to answer questions and withdraw from the interview at any time. Most FGD participants spoke Georgian and some Russian. Most FGDs were held in Georgian, some in Russian, and some in dual languages. The facilitator was fluent in Georgian and Russian, whilst the interpreter was proficient in English and Georgian and could fully comprehend Russian. In the FGDs simultaneous translation to English was done, and individual interviews were translated sentence by sentence. The translations were recorded on audiotape as back-up but not transcribed verbatim. Detailed notes were taken throughout the fieldwork.

The topic “Local disease panorama and disease priorities” was explored in two steps. First, participants were asked to mention all diseases or health problems they had experienced in their small ruminant herds during the last three years (i.e. including the PPR outbreak in 2016). Following this first step, participants were asked to prioritise five of the mentioned health problems that were most important for them. If any diseases or syndromes resembling PPR were mentioned in the first or second step these were discussed further to confirm or exclude occurrence of PPR. If PPR was confirmed, the epidemiology was further investigated using seasonal calendar and proportional piling tools, as described by e.g. Jost et al. (2007) to appreciate herd morbidity and mortality [64].

Notes from the FGDs were compiled into one master note per interview and summarised. Data from the additional interviews and informal talks with key informants were used to contrast and compare the results.

## Results

### General summary of sampling

**Fig. 2:**
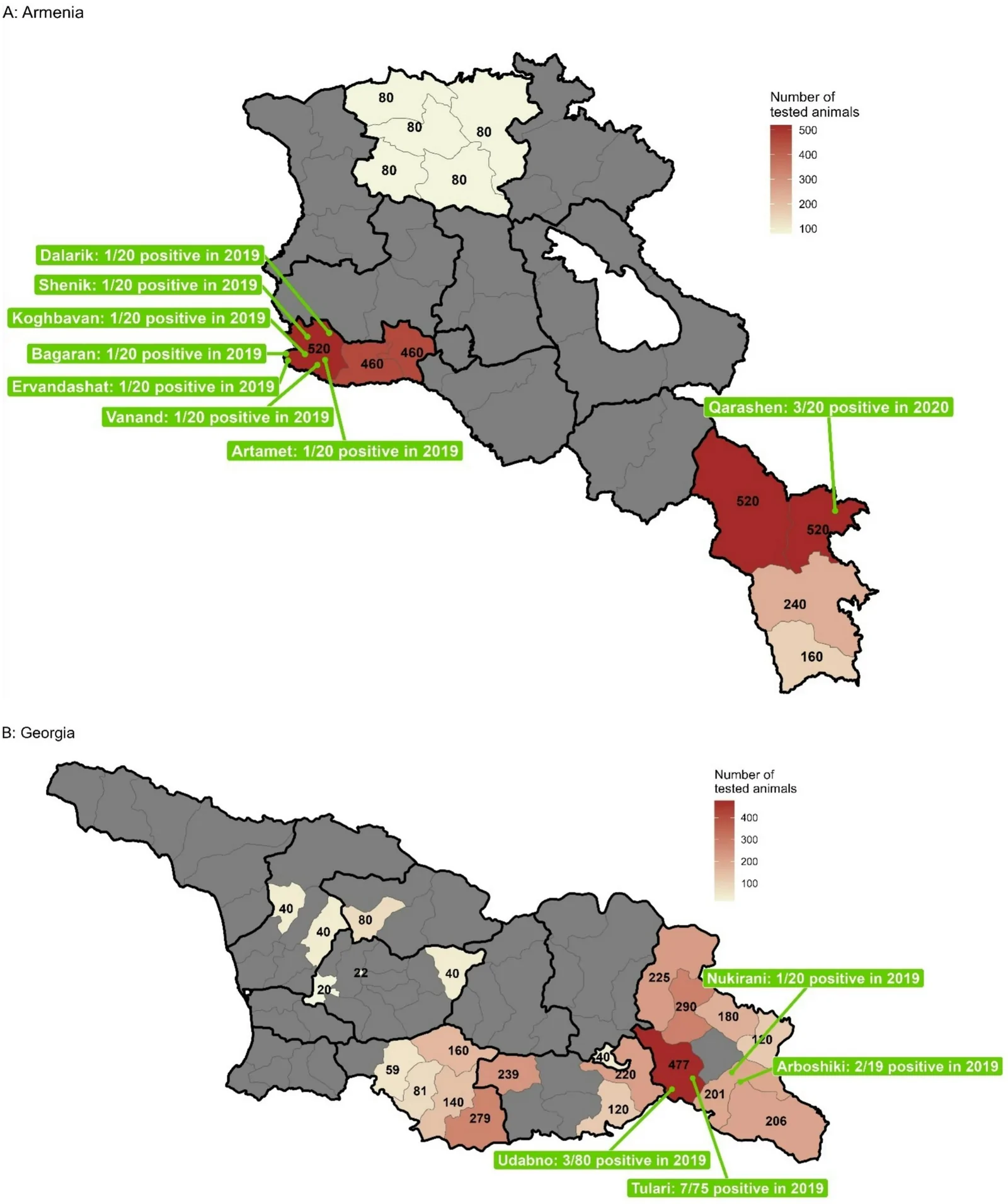
Distribution of samples at the municipality level in Armenia (**A**) and Georgia (**B**) from a sero-surveillance study for PPR conducted in 2019–2020. The locations of seropositive animals are shown in green, aggregated at the community level

Overall, 3,280 animals were sampled over two years in Armenia (1,640 per year) and 3,279 in Georgia (1,600 in 2019 and 1,679 in 2020) for a total of 6,559 animals (Table 2). Sampling was conducted in 3 of 11 provinces of Armenia and in 7 of 10 provinces of Georgia. These provinces had an approximate combined population of 232,000 small ruminants in Armenia and 811,000 in Georgia. In these provinces, 99 of 319 communities were selected in Armenia (representing all 12 municipalities) and 22 of 47 municipalities in Georgia, containing 177,000 and 690,000 small ruminants respectively (Table 2; Fig. 2, supplementary tables SP1 and SP2). Of 99 Armenian communities, 65 were sampled both years, with 17 being sampled in 2019 alone, and another 17 having in 2020 alone, because the initial 17 no longer qualified with population size or herd number requirements for sampling.

**Table 2:** Summary of sampling by province in Armenia and Georgia for the 2019–2020 period

	Number of units* (Number sampled)	Total small ruminant population of province	Small ruminant population in sampled municipalities	Number of samples
Armenia	319 (99)	231,638	177,132	3,280
Armavir	96 (45)	102,788	85,804	1,440
Lori	115 (10)	25,579	4,028	400
Syunik	108 (44)	103,271	87,300	1,440
Georgia	47 (22)	810,867	690,268	3,279
Kakheti	8 (7)	490,976	475,952	1,699
Kvemo Kartli	7 (3)	212,864	127,063	579
Samtskhe Javakheti	6 (5)	79,348	79,147	719
Tbilisi**	1 (1)	0	0	40
Imereti***	12 (3)	21,280	4,185	82
Racha Lechlum K/S	4 (1)	222	169	80
Samegrelo Z/S	9 (2)	6,177	3,752	80

*Unit refers to the community (third-order administrative division) in Armenia, and the municipality (second-order administrative division) in Georgia

**Tbilisi did not have a recorded population in the population census data. However, it had a reported population of 7,790 in a published study [40]. This number was used for analysis but is not included in the table

***One of the three sampled municipalities in Imereti, the city of Kutaisi, had no reported small ruminant population in the population data provided. For modelling purposes, it was assumed that the population not captured by the other two sampled municipalities (17,095) was in the catchment area of Kutaisi and used as Kutaisi’s population. Given the small sample size in that municipality relative to population, this is very close to the assumption of infinite population that would have been used if we ignored population numbers

### Laboratory results for serum and blood sample

In Armenia, a total of 10 serological samples were positive. Of these, seven occurred in 2019 in seven different communities in Baghramyan municipality, Armavir province (one per community), none of which had seropositive cases when sampled in 2020. Another three occurred in 2020 in one community of Syunik province (municipality of Goris), which had no seropositive when sampled in 2019. In Georgia, 13 serological samples were positive. All of these occurred in 2019, in three municipalities of Kakheti province (Dedoplistskaro, Sagaredjo, and Signagi). Two occurred in one community of Dedoplistskaro municipality, one in a community of Signagi municipality, and three and seven respectively in two communities of Sagaredjo municipality (Fig. 2). No blood sample was positive for viral RNA; thus, no evidence of virus presence was detected. As serological samples were sporadic and not spatially clustered, and with no evidence of virus presence, this suggested absence of active PPRV circulation. Furthermore, in the presence of sero-positive animals, in Georgia, NFA conducted follow-up investigations in the regions where serologically positive samples had been investigated. In farms where animals were still present at the time of the visit, repeat sampling was carried out. All sero-positive animals were observed and clinically investigated. None of them had showed clinical signs of PPR. However, in a number of farms, the animals had already been sold, which made re-sampling impossible. The presence of the few sero-positive animals could be explained by trade and movements of previously vaccinated or exposed animals, and animal owners explained that animals were purchased without any records.

### Disease freedom calculations

**Fig. 3:**
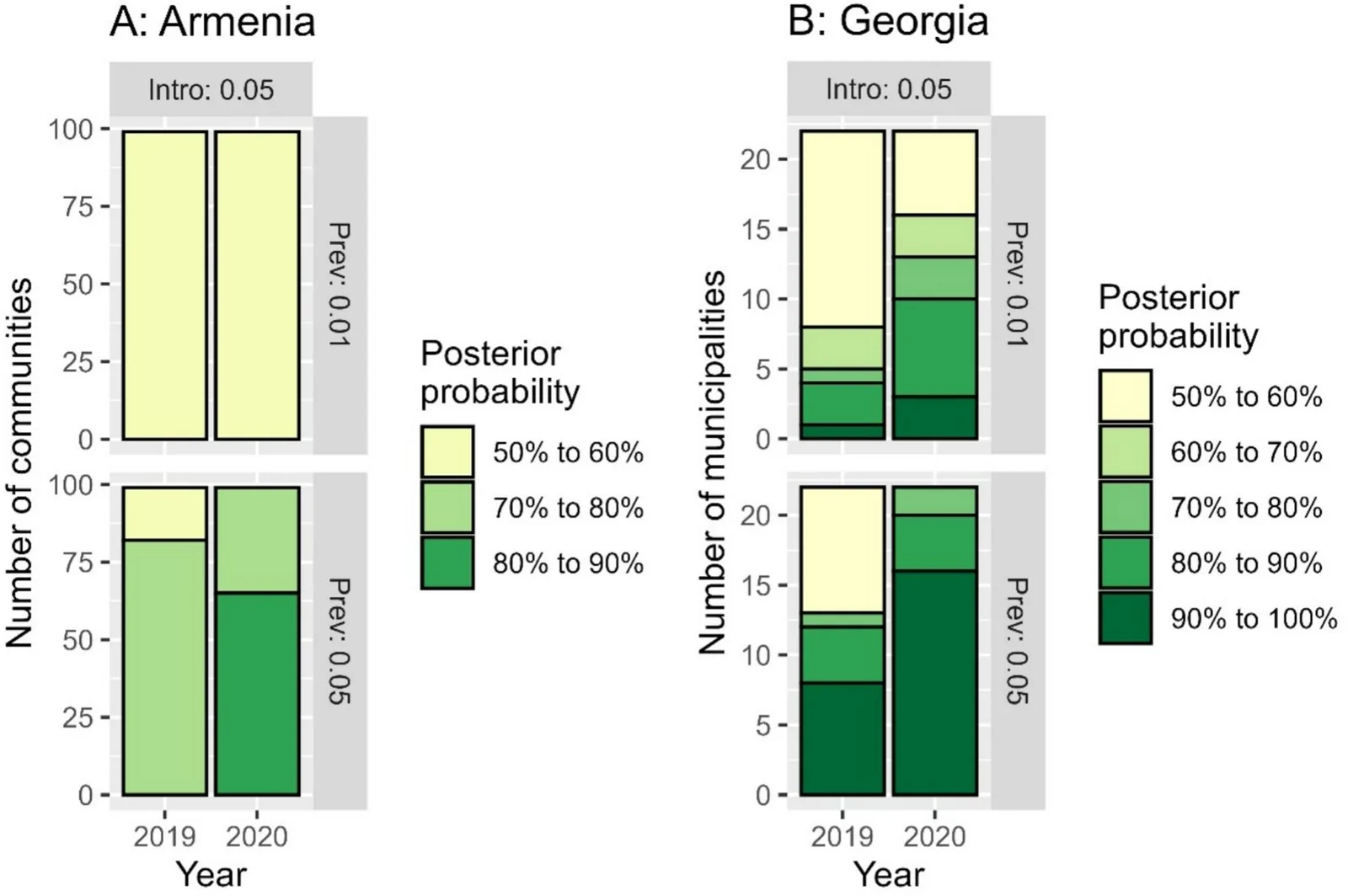
Distribution of sampling units by probability of disease freedom under different parameter value combinations. “Prev” are values of design prevalence, and “Intro” are values of probability of introduction. Results for Armenia are on panel **A**, and for Georgia on panel **B**

With a yearly probability of introduction of 5% and a design prevalence of 1%, the median unit level posterior probability of disease freedom was of 58.9% in Armenia and 85.9% in Georgia (Table 3). We observed very little variation when changing the probability of introduction at the unit level. Changing the design prevalence, however, led to important changes in unit level probabilities of disease freedom. When increasing the design prevalence to 5%, the median value reached 85.9% in Armenia and 99.7% in Georgia. In Armenia, none of the units reached values above 60% in 2020 with the 1% design prevalence, but 34 units reached values between 70% and 80% and the remaining 65 between 80% and 90% with the 5% design prevalence. For Georgia, 10 units had values above 80% with the 1% design prevalence compared to 20 units with the 5% design prevalence (Fig. 3). This distribution did not change with different values of probability of introduction.

**Table 3:** Probabilities of disease freedom in the year 2020 at the unit level (second year of sampling) for different parameter values in Georgia and Armenia

Design prevalence		Armenia		Georgia	
	Probability of introduction	Median	Mean	Median	Mean
1%	1%	58.94%	57.51%	76.37%	75.43%
1%	5%	58.92%	57.49%	76.30%	75.34%
1%	10%	58.89%	57.46%	76.22%	75.23%
5%	1%	85.91%	81.11%	99.69%	94.37%
5%	5%	85.90%	81.09%	99.66%	94.30%
5%	10%	85.88%	81.07%	99.54%	94.21%

At the national level, values between Armenia and Georgia were more similar, with a disease freedom value of 70.8% in Armenia, and 73.8% in Georgia when using a design prevalence of 5% and a probability of introduction of 5% (Table 4). At this level, the impact of changing design prevalence was noticeable on the 2020 values for disease freedom, though to a lesser extent than observed at the unit level. Increasing the probability of introduction led to a more noticeable decrease in probability of freedom compared to the unit level.

**Table 4:** Posterior probability, equilibrium value, and time to reach 90% confidence of disease freedom at the national level. PDF=probability of disease freedom

Design prevalence		Armenia 2020			Georgia 2020		
	Probability of introduction	PDF	Equilibrium	Years to 90%	PDF	Equilibrium	Years to 90%
1%	1%	56.5	93.3	22	66.2	97.8	5
1%	5%	54.4	65.4	NA	64.1	88.4	NA
1%	10%	51.7	26.8	NA	61.4	75.6	NA
5%	1%	72.9	98.5	3	75.8	98.8	2
5%	5%	70.8	92.1	6	73.8	93.9	4
5%	10%	68.0	83.2	NA	71.1	87.0	NA

**Fig. 4:**
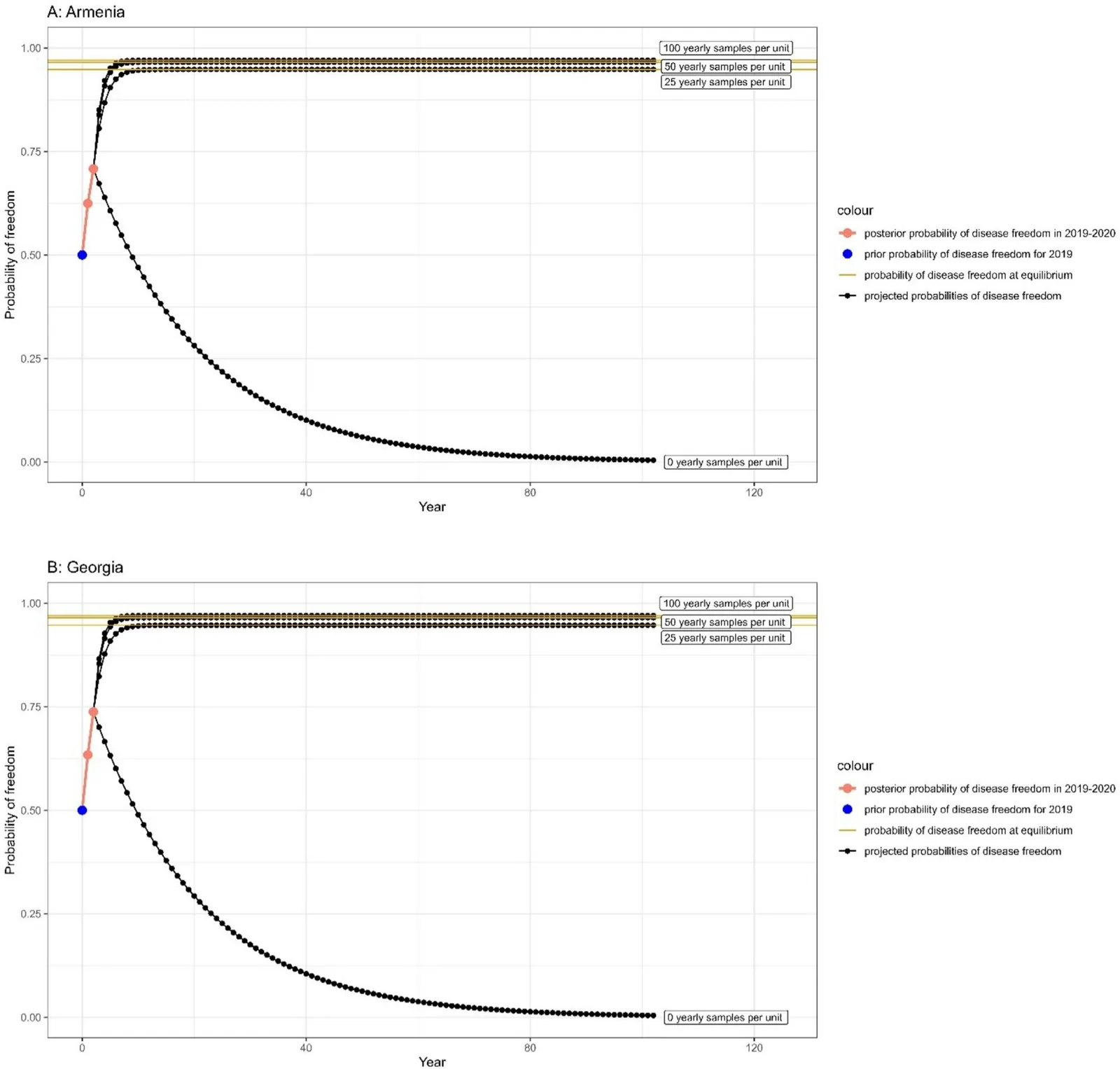
Evolution of the probability of disease freedom over 100 years with different sampling scenarios and with a design prevalence of 5% and probability of introduction of 5% in Armenia (**A**) and Georgia (**B**). The blue point is the default assumption for prior probability of 50% for 2019, pink points represent actual values calculated for 2019 and 2020, and black points represent projections with sampling of 0, 25, 50, or 100 animals per unit and year

As seen in Fig. 4, a high probability of disease freedom, above 90%, would be reached in a short time, with the maximum of three years observed for Armenia with a sampling of 25 animals per unit and year, a design prevalence of 5% and a probability of introduction of 5%, regardless of the sampling scenario (25, 50, or 100 animals per unit and year). When projecting towards equilibrium and with the fixed sampling scenarios, Armenia reached higher probability of disease freedom than Georgia, despite having lower values for 2020. As sampling stopped after 2020, we also projected how national values would change in the absence of further sampling, which leads to a quick drop of probability of freedom: close to 60% by 2024 for both countries, and below 50% by 2027 in Armenia, and 2028 in Georgia (Fig. 4). Even with the higher probability of introduction of 10%, an equilibrium of 89% for the probability of disease freedom was achieved with 25 samples per unit and year. However, reducing the design prevalence to 1% lead to much lower equilibrium values, although usually above the values calculated for 2020. The only parameter variation where the probability of freedom dropped despite continued sampling was for Georgia with a design prevalence of 1%, a probability of introduction of 10%, and a sampling frame of 25 animals per unit and year. In this case, equilibrium was at 51%, well below the value of 74% observed in 2020.

### Participatory disease surveillance

Twenty-seven FGDs were performed with a total of 149 participants, including 47 women and 102 men. In addition, 14 supplementary interviews were done. All participants owned or cared for small ruminants, either themselves or within the family. Flocks frequently consisted of both sheep and goats, but with a dominance of sheep.

While exploring the topic “Local disease panorama and disease priorities” a total of 64 different problems (mostly diseases, but also some problems related to small ruminant management) were mentioned, ranging from 6 to 18 problems per FGD [40]. PPR was never mentioned, including in FGDs with participants that had been affected by the outbreak in 2016. Mentions of diseases commonly recognized as differential diagnoses for PPR such as stomatitis, rhinitis, and diarrhoea were further investigated focusing on clinical signs and epidemiological parameters such as morbidity, mortality and perceived contagiousness. In all these instances suspicions of PPR could be excluded. Most of the participants reported having heard of the PPR outbreak in 2016, whereas none had heard of, or experienced, any other outbreaks. Participants in one FGD from the Tbilisi province with participants that had been affected by PPR in January 2016 described a serious disease affecting all herds in the community with results from proportional piling indicating high morbidity (59%), mortality (44%) and case fatality rate (75%) at the community level.

## Discussion

The results from the sero-surveillance in 2019–2020 in Armenia and Georgia detected no evidence for PPRV circulation in either country. Very few serological samples returned positive and these where not spatially clustered. Furthermore, none of these animals tested positive for viral RNA. After further investigation in animals that could be identified again, none showed clinical signs. This is similar to a study conducted in Lao where very low sero-prevalence in the absence of clinical signs of PPR was used as evidence of absence of PPRV circulation [65]. This was further corroborated in our study, by the addition of viral RNA testing in blood which returned no positive samples. The presence of the few sero-positive animals could be explained by trade and movements of previously vaccinated or exposed animals as well as possible false positive tests. With the sampling framework used, this provided moderate evidence of national PPRV freedom, varying from 52% to 73% in Armenia, and 61% to 76% in Georgia depending on the parametrization of the model. It is important to note, that as the sampling focused on subset of each country considered at higher risk, and not full geographical coverage, that the so-called national values of freedom are, strictly speaking, valid within the geographical subset that was sampled. However, as unsampled areas were either similar or could be considered at lower risk of introduction, thanks to greater distance from endemic and outbreak areas or had a lower small ruminant population, we can assume that the result observed here would be similar for the rest of each country had it been sampled. At the unit level, we observed much higher probabilities of freedom in Georgia compared to Armenia. This can be explained by the difference in the level at which data was aggregated. In the model Georgia had fewer and larger units (municipalities) with a larger and more variable unit sample sizes. Armenia had a large number of small units (community) with a fix sample size of 20 animals per year. Thus, Georgian units had on average larger unit level-sensitivity values and thus freedom values. At the national level these differences between countries were less important as both countries had a similar overall sampling design in place with the same sample size. The Armenian national values remain lower as they are mathematically penalized by having more small units in the model, even though this gives a more precise and accurate description of the system. The main factor preventing obtaining higher values was the short time period of active surveillance, especially in areas at high risk of introduction. This short time period also explains why the different values of probability introduction showed little differences in results, as there was not enough time to see the cumulated discounting effect of introduction on disease freedom probabilities. Surveillance programs that have achieved high probabilities of disease freedom have typically been conducted over multiple years, with sustained and homogeneous levels of sampling [66–68]. Projections in our study showed that if the surveillance framework used in Armenia and Georgia had been sustained, 90% or higher probability of freedom could theoretically have been achieved within two to five years under certain model parametrizations. Even with a relatively small sample size per unit, a surveillance system could over time reach high levels of confidence in disease absence. This is especially true when targeting high-risk animals, which was not explicitly modelled here, as this increases the sensitivity of the surveillance system [56]. It is, however, important to note that the sampling frame in this study was not designed as a long-term surveillance framework for disease freedom calculations. The study was designed to quantify circulation of PPRV and provide point-in-time estimates for sero-prevalence. The decision to use the data for freedom calculation was taken after sampling and analysis as it became clear that no evidence of PPRV circulation was detected. Risk-based cluster-based sampling as performed in this study is recommended for disease freedom calculations [54]. However, if a study or surveillance system aims to demonstrate high probabilities of disease freedom it is important to both maximize the surveillance sensitivity of the system being studied and, because of the temporal discounting, provide sustained sampling over long periods of time. Surveillance sensitivity can be improved by ensuring a well-balanced sampling strategy, with clusters (ideally herds) being properly recorded. This can also be improved by sampling higher risk animals whilst recording their risk profile to be able to apply scenario tree modelling in which higher-risk animals have higher individual disease detection probability [56, 57]. The lack of long-term sampling, and risk-profile information to implement scenario-tree modelling were two key limitations of the surveillance data used in this study in the context of disease freedom calculations. The long-term evaluation of disease freedom was also limited by the lack of quantitative data on risk of introduction which led us to use a simple assumption for how this risk is distributed spatially. Furthermore, in the case of Georgia, sampling data did not include the sampling cluster details which limited the resolution of the analysis.

Small sample sizes as well as unbalanced or irregular sampling limit the usefulness of sero-surveillance, especially if the goal is to demonstrate freedom [65, 69]. Financial and operational constraints in the animal health sector, frequent in low- and middle-income countries, influence these aspects of surveillance planning and implementation. A common challenge in both countries, experienced in this study, is the process of keeping an updated, reliable and detailed animal census. Yearly migrations and formal and informal animal movements cause a rapid population turnover and changes in size and composition, which might not be recorded in a detail regularly. This in turns made it difficult to strictly adhere to the designed sampling frame as communities in both countries which initially met sampling criteria had to be replaced later as they did not meet these anymore. Similar limitations were observed in a study in Lao [65]. This is also an issue at the individual animal level, as animals are not individually registered, making it possible to accidently sample animals outside of the sampling criteria (based on age and vaccination history). In this study we further had difficulties in obtaining recent information on test performance, specifically in non-endemic settings, with the test evaluation having been conducted more than 30 years ago [49]. Nevertheless, our study still provides moderate evidence that PPRV was not circulating at the time of sampling in both countries. In Georgia this claim was additionally strengthened by the results from the participatory disease surveillance that, like the sero-surveillance, provided no evidence of PPRV circulation. This confirms that participatory disease surveillance can be a valuable complementary or alternative component not only for disease surveillance but also for estimating freedom from disease. Indeed, participatory methods were an important part in especially the last phase of the global eradication campaign for Rinderpest [70–72].

As can be seen from the recent spread of PPR in the previously free Balkan region, the emergence in Georgia in 2016 and the re-emergence in 2024, non-endemic countries remain vulnerable to introduction, especially when bordering countries where the disease is endemically present. This is especially true in the presence of porous borders, informal transboundary trade in domestic ruminants, or transboundary wild ruminant populations and habitats. Cooperation between neighbouring countries in different stages of the WOAH four-stage PPR-eradication strategy is thus necessary for ensuring continued progress towards global eradication [16]. The Caucasus region is a key example of needs, opportunities, and challenges in this regard. The need for regional cooperation can be seen in the presence of countries at different stages: from the first control stage to PPR-free countries with both susceptible domestic and wild species in large numbers.

Recent conflicts in the region and in the neighbouring Middle East have involved directly or indirectly every country in the Caucasus, leading to political and economic instability as well as mistrust between countries including on topics such as animal health and trade. This study thus demonstrates a case of countermovement to this negative trend, a successful cooperation between two neighbouring countries regarding an important transboundary pathogen. The new outbreak in Georgia in 2024 confirms the continued high risk of introduction for countries in the Caucasus region, the need for cooperation and surveillance, and for control systems that are able to rapidly detect and respond to introductions.

## Supplementary Information

Supplementary Material 1: Supplementary document 1: topic guide for participatory disease surveillance interviews. Supplementary table SP1: sampling summary at the municipality level for Armenia. Supplementary table SP2: sampling summary at the municipality level for Georgia.

## Data Availability

The data is available upon reasonable request and is subject to the approval of the governmental institutes in Georgia and Armenia that participated in this study: LPEL State Laboratory of Agriculture of Georgia, LEPL National Food Agency of Georgia, and Scientific Centre for Risk Assessment and Analysis in Food Safety Area of Armenia.
