# Supplementary material for: Serosurveillance and participatory disease surveillance of Peste des Petits Ruminants (PPR) in Georgia and Armenia in 2019–2020
Source: BMC Vet Res. 2026 Sep 4;22:516. doi: 10.1186/s12917-026-05781-5 (PMC13545762; doi:10.1186/s12917-026-05781-5)
Supplement: Supplementary file 1 [file 12917_2026_5781_MOESM1_ESM.docx]

*Supplementary document 1: topic guide for participatory disease surveillance interviews.*

# PPR Caucasus

## Interview/topic guide

### Warm up

Do you have/own/take care of sheep and goats?

### Management

How are these sheep and goats managed?

Together draw a timeline with the important shoat-year-events (

|  | Year | | | | | | | | | | | |
| --- | --- | --- | --- | --- | --- | --- | --- | --- | --- | --- | --- | --- |
|  | J | F | M | A | M | J | J | A | S | O | N | D |
| Timeline events |  | lambing | lambing |  |  |  |  |  |  |  |  |  |
| Disease outbreaks in herd: |  |  |  |  |  |  |  |  |  |  |  |  |
| Disease outbreaks that participants think were PPR (if any) |  |  |  |  |  |  |  |  |  |  |  |  |

### Disease perception

How is the sheep and goat farming going?

Do you have problems with disease in sheep and goats?

Which disease have you encountered in the sheep and goats in the last three

years?

Of these, which five diseases do the group consider most important, and why?

Facilitator and researchers together pick out the ones that are respiratory/gastric/viral=could be PPR and explain the process to the group. For each of these selected diseases:

How does this disease show on the sheep or goat?

When you see those signs are you sure that it is that specific disease?

What is causing the disease?

Why/why not is this disease (not) important for you?

### Disease management

What do you do when an animal gets sick? (Poke for details, do not accept only “I call the vet”)

What do you do to protect/prevent your sheep and goats from getting sick?

### Seasonal calendar

Do you remember instances in the past when many animals got sick at the same time? What do you think was the sickness?

When did these outbreaks occour in relation to the shoat management timeline? Illustrate by scoring occurrence using the timeline that have been produced, and 100 counters for each question. Use the timeline produced for one Q at time, take photos and record the numbers according to how the timeline looks. (example below)

Note all discussions!

### PPR outbreaks

Describe outbreaks that the participants or the research team (based on answers from questions above) think were PPR.

For each outbreak: proportion of owners in their village/epidemiological unit affected (=had at least one sick shoat) or not affected, proportion of sheep and goats that were sick and died, were sick but recovered, or not affected at all. Scores made by proportional piling separately for farmer/shoats and for each outbreak.

**Data capture:**

| Outbreaks | Date (month/year) | Owners (proportional piling) | | Animals (proportional piling) | | |
| --- | --- | --- | --- | --- | --- | --- |
|  |  | Affected | Non-affected | Dead | Recovered  (sick but survived) | Healthy  (never sick) |
| Outbreak 1 |  |  |  |  |  |  |
| Outbreak 2 |  |  |  |  |  |  |
| Outbreak 3 |  |  |  |  |  |  |
| Outbreak 4 |  |  |  |  |  |  |

### PPR outbreak impact

What was the consequences of these outbreaks (including impact of related control measures)?

### Rounding up

Anything else you would like to discuss?

*Supplementary table SP1: sampling summary at the municipality level for Armenia*

| **Province** | **Municipality** | **Communities**  **2019** | **Samples**  **2019** | **Communities**  **2020** | **Samples**  **2020** |
| --- | --- | --- | --- | --- | --- |
| **Armavir** | **3 municipalities** | **36** | **720** | **36** | **720** |
|  | Armavir | 12 | 240 | 11 | 220 |
|  | Baghramyan | 12 | 240 | 14 | 280 |
|  | Vagharshapat | 12 | 240 | 11 | 220 |
| **Lori** | **5 municipalities** | **10** | **200** | **10** | **200** |
|  | Gugark | 2 | 40 | 2 | 40 |
|  | Spitak | 2 | 40 | 2 | 40 |
|  | Stepanavan | 2 | 40 | 2 | 40 |
|  | Tashir | 2 | 40 | 2 | 40 |
|  | Tumanian | 2 | 40 | 2 | 40 |
| **Syunik** | **4 municipalities** | **36** | **720** | **36** | **720** |
|  | Goris | 13 | 260 | 13 | 260 |
|  | Kapan | 6 | 120 | 6 | 120 |
|  | Meghri | 4 | 80 | 4 | 80 |
|  | Sisian | 13 | 260 | 13 | 260 |
| **Armenia** | **12 municipalities** | **82** | **1640** | **82** | **1640** |

*Supplementary table SP2: sampling summary at the municipality level for Georgia*

| **Province** | **Municipality** | **Communities**  **(design)** | **Samples**  **(design)** | **Samples**  **2019** | **Samples**  **2020** |
| --- | --- | --- | --- | --- | --- |
| **Imereti** | **3 municipalities** | **1** | **40** | **40** | **42** |
|  | Kutaisi | 0 | 0 | 0 | 22 |
|  | Sachkhere | 1 | 40 | 40 | 0 |
|  | Samtredia | 0 | 0 | 0 | 20 |
| **Kakheti** | **7 municipalities** | **21** | **860** | **863** | **836** |
|  | Akhmeta | 0 | 0 | 0 | 225 |
|  | Dedoplistskaro | 7 | 280 | 206 | 0 |
|  | Kvareli | 0 | 0 | 0 | 180 |
|  | Lagodekhi | 0 | 0 | 0 | 120 |
|  | Sagaredjo | 10 | 420 | 477 | 0 |
|  | Signagi | 4 | 160 | 180 | 21 |
|  | Telavi | 0 | 0 | 0 | 290 |
| **Kvemo Kartli** | **3 municipalities** | **7** | **420** | **399** | **180** |
|  | Gardabani | 4 | 240 | 220 | 0 |
|  | Marneuli | 2 | 120 | 120 | 0 |
|  | Tsalka | 1 | 60 | 59 | 180 |
| **Racha Lechkhum K/S** | **1 municipality** | **1** | **40** | **40** | **40** |
|  | Tsageri | 1 | 40 | 40 | 40 |
| **Samagrelo Z/S** | **2 municipalities** | **1** | **40** | **40** | **40** |
|  | Martvili | 1 | 40 | 40 | 0 |
|  | Tsalenjikha | 0 | 0 | 0 | 40 |
| **Samtskhe Javakheti** | **5 municipalities** | **4** | **160** | **178** | **541** |
|  | Akhalkalaki | 0 | 0 | 0 | 140 |
|  | Akhaltsikhe | 0 | 0 | 19 | 40 |
|  | Aspindza | 0 | 0 | 0 | 81 |
|  | Bordjomi | 2 | 80 | 80 | 80 |
|  | Ninotsminda | 2 | 80 | 79 | 200 |
| **Tbilisi** | **1 municipality** | **1** | **40** | **40** | **40** |
|  | Tbilisi | 1 | 40 | 40 | 0 |
| **Georgia** | **22 municipalities** | **36** | **1600** | **1600** | **1679** |
